# Development and validation of a computer program for measuring emotional awareness in German—The geLEAS (German electronic Levels of Emotional Awareness Scale)

**DOI:** 10.3389/fpsyt.2023.1129755

**Published:** 2023-03-23

**Authors:** Julian Herpertz, Jacob Taylor, John J. B. Allen, Stephan Herpertz, Nils Opel, Maike Richter, Claudia Subic-Wrana, Jan Dieris-Hirche, Richard D. Lane

**Affiliations:** ^1^Institute for Translational Psychiatry, University of Münster, Münster, Germany; ^2^David A. Dunlap Department of Astronomy and Astrophysics, University of Toronto, Toronto, ON, Canada; ^3^Department of Psychology, University of Arizona, Tucson, AZ, United States; ^4^Department of Psychosomatic Medicine and Psychotherapy, LWL-University Hospital, Ruhr University Bochum, Bochum, Germany; ^5^German Center for Mental Health (DZPG), Site Jena-Magdeburg-Halle, Jena, Germany; ^6^Center for Intervention and Research on Adaptive and Maladaptive Brain Circuits Underlying Mental Health (C-I-R-C), Jena-Magdeburg-Halle, Jena, Germany; ^7^Department of Psychiatry and Psychotherapy, Jena University Hospital, Friedrich-Schiller-University Jena, Jena, Germany; ^8^Department of Psychosomatic Medicine and Psychotherapy, University Hospital Cologne, University of Cologne, Cologne, Germany; ^9^Departments of Psychiatry, Psychology and Neuroscience, University of Arizona, Tucson, AZ, United States

**Keywords:** levels of emotional awareness, alexithymia, computerized assessment, emotion assessment, performance scale, emotions, text analysis

## Abstract

**Introduction:**

Emotional awareness is the ability to identify, interpret, and verbalize the emotional responses of oneself and those of others. The Levels of Emotional Awareness Scale (LEAS) is an objective performance inventory that accurately measures an individual's emotional awareness. LEAS assessments are typically scored manually and are therefore both time consuming and cognitively demanding. This study presents a German electronic scoring program for the LEAS (geLEAS), the first non-English computerized assessment approach of the LEAS.

**Methods:**

Data were collected from a healthy German community sample (*N* = 208). We developed a modern software for computerizing LEAS scoring, an open-source text-based emotion assessment tool called VETA (Verbal Emotion in Text Assessment). We investigated if the software would arrive at similar results as hand scoring in German and if emotional awareness would show similar associations to sociodemographic information and psychometric test results as in previous studies.

**Results:**

The most frequently used scoring method of the geLEAS shows excellent internal consistency (α = 0.94) and high correlations with hand scoring (*r* = 0.97, *p* < 0.001). Higher emotional awareness measured by the geLEAS is associated with female gender, older age, and higher academic achievement (all *p* < 0.001). Moreover, it is linked to the ability to identify emotions in facial expressions (*p* < 0.001) and more accurate theory of mind functioning (*p* < 0.001).

**Discussion:**

An automated method for evaluating emotional awareness greatly expands the ability to study emotional awareness in clinical care and research. This study aims to advance the use of emotional awareness as a clinical and scientific parameter.

## 1. Introduction

Emotional awareness is the ability to identify, interpret, and verbalize the emotions of oneself and of others (1, 2). It is a socio-emotional skill that may be a precondition for the use of adaptive emotion regulation strategies [e.g., acceptance, problem solving, reappraisal (3)]. Hence, it is clinically relevant to the vulnerability to and maintenance of mental disorders (4). Low emotional awareness has been associated with borderline personality disorder (5), somatoform disorder (6), eating disorders (7), depression (8, 9), schizophrenia (10), and substance abuse (11). Therefore, a method for quantifying and thus objectifying emotional awareness is of great clinical and scientific interest.

Lane and Schwartz developed the Levels of Emotional Awareness Scale (LEAS) (12), a performance scale for the measurement of emotional awareness. Derived from Piaget's model of cognitive developmental stages (13), the LEAS assesses five levels of emotional awareness: bodily sensations [1], undifferentiated emotions or action tendencies [2], single explicit emotions [3], blends of emotions [4], and blends of blends of emotions [5] (See Table 1). In contrast to the stages of cognitive development, which were thought to emerge sequentially during childhood, the levels of emotional awareness are functional forms of organization that have a developmental progression but can shift in either direction at any time (2). A LEAS questionnaire presents 20 hypothetical, emotion evoking situations to its subjects and asks the respondent how both themselves and a counterpart would feel in the presented situation. For example: “Your boss tells you that your work has been unacceptable and needs to be improved. How would you feel? How would your boss feel?.” Subjects then write down their answers in an open-ended text format. With the help of an extensive word-list and a detailed scoring manual, a trained scorer then assigns the responses to one of the five levels (See Table 2). Each respondent's protocol consists of 20 emotion evoking questions called items. The highest level (level five) of emotional awareness is linked to blends of emotions in oneself and the other person (e.g., “I would feel angry and disappointed, he would feel frightened and ashamed.”). Since its implementation, the LEAS has been used in numerous studies and there is strong evidence for the construct validity of the LEAS as a measure of emotional awareness (2, 14, 15). The LEAS has also been proposed as a diagnostic tool for alexithymia. Literally meaning “no words for mood,” alexithymia describes a personality construct that is characterized by an impaired ability to be aware of, explicitly identify, and describe one's feelings (16). It is thought to be caused by an arrest on the developmental continuum of emotional awareness (17). Consistent with findings linking mental disorders with lower emotional awareness, greater degrees of alexithymia have been associated with eating disorders (18, 19), personality disorders (20), and major depressive disorder (21, 22). Moreover, an association between alexithymia and suicidal ideation, non-suicidal-self-injury, and an increased mortality rate has been observed (23, 24).

**Table 1:** Comparing the Lane/Schwartz and Piaget models.

**Stages**	**Lane/Schwartz**	**Piaget**
1	“My heart would race”	
	(Bodily sensation)	Concrete representations
2	“I would cry”	(Sensor-motor)
	(Action tendency)	
3	“I would feel sad” (Explicit emotion)	Language and thinking (Pre-operational)
4	“I would feel sad but at the same time relieved”	Use of logic (Concrete operational)
	(Blends of emotions)	
5	“I would feel sad but at the same time relieved	Deductive reasoning (Formal operational)
	My partner feels surprised and guilty”	
	(Blends of blends of emotions)	

**Table 2:** The five levels of emotional awareness example.

**Level**	**Description**	**Example response**
0	Cognitions	I would think he was wrong.
1	Bodily sensations	I would have a severe headache.
	Action tendencies	I would cry, because he disapproves of my work.
2	Non-specific valenced	Both of us would feel bad.
	emotions	
3	Single specific emotion	I would feel sad.
4	Blends of emotions	I would feel sad and angry about myself.
5	Combinations of blends	I would feel sad and angry. He would be disappointed and wants me to do better

Scenario: “Your boss tells you that your work has been unacceptable and needs to be improved.

How would you feel? How would your boss feel?”

There are three reasons why a performance measure like the LEAS might be superior to self-report measures like the Toronto Alexithymia Scale (TAS-20) for the purpose of evaluating alexithymia:

1. Self-reports ask subjects to evaluate their ability to identify and verbalize their emotions. If a person has difficulties conceptualizing and verbalizing their emotional state, it is not clear whether he or she is able to accurately reflect on this difficulty in a questionnaire (14). The LEAS does not rely on the subject's assessment but measures emotional awareness directly in terms of the response to an emotion-provoking scenario. LEAS scores can then be compared to those of control groups or unpublished norms in healthy volunteers.
2. In contrast to self-report measures, lower emotional awareness measured by the LEAS does not correlate with negative affect (25, 26). As elevated negative affect itself is often related to mental disorders, it is important that assessment of impairment in the ability to identify and report on negative affect is independent of reported negative affect.
3. Self-report measures such as the TAS-20 only define a continuum of impairment as they assess the absence or the presence of alexithymic traits. The LEAS can identify limitations in emotional awareness corresponding to alexithymia. Moreover, it can detect high levels of emotional awareness beyond the absence of impairment which have been suggested to be contributing to stress resilience (2, 14).

Based on the aforementioned considerations, the LEAS has the potential to play a key role in mental health care and science; however, manual scoring requires significant time and effort (27). Hence, only few people can contribute to the use of the LEAS in research and mental health care, resulting in an often denied access to important patient information. A computerized evaluation method that automatically scores LEAS protocols is a cost-efficient, time, and labor-saving alternative to hand scoring. While the LEAS has been translated into 16 different languages [Arabic, Brazilian Portuguese, Chinese, Croatian, Czech, Dutch, French, German, Greek, Hebrew, Italian, Japanese, Korean, Persian, Portuguese, and Spanish (2)], computerized scoring to date is only possible in English. Barchard et al. (15) introduced the Program for Open-Ended Scoring (POES), a software program designed for automated LEAS scoring. POES relies on two inputs: the respective items of the subjects and a word list with an assigned value for each word or phrase that reflects emotional awareness. The program can identify a word in an item, retrieve it in the word list and assign the respective value in the output file. POES arrives at similar scoring results as hand scoring and was successfully implemented to score the eLEAS [e for electronic (15)].

This study presents the first non-English computerized assessment of the LEAS, the German electronic Levels of Emotional Awareness Scale (geLEAS). In addition to providing experimental results evaluating the effect of computerized LEAS assessment for the German language, we present a new open source, text-based emotion assessment tool called VETA (Verbal Emotion in Text Assessment). VETA makes use of many of the modern *Python* tools that have been popularized since POES's inception in 2010. Written in *Python 3* with a focus on Object-Oriented design, VETA implements a class structure based on the nature of a typical LEAS assessment. All of the analyses for this report were completed in *Python*. VETA's source code will contain *Python* notebooks with examples of how to use the program. We plan to add the package to the Python Package Index (PIP) for ease of installation.

A recent study using the manual French version of the LEAS reported that higher levels of educational attainment (measured in years of training) and female gender was associated with higher emotional awareness (28) in the French population. The study replicated findings by Lane et al. (29) on the association of emotional awareness and education as well as findings by Barret et al. (30) on the association of gender with emotional awareness. Furthermore, it was shown that a more accurate perception of emotions in facial expressions is correlated with a better performance in the manual LEAS (14). Lastly, it was recently found that a better ability to infer mental states in other people (theory of mind) is closely linked to a higher emotional awareness measured by the LEAS (31).

In order to assess the validity of the geLEAS, we collected LEAS data from a healthy German community sample. We had both trained raters and VETA score the protocols and investigated if both scoring procedures generated similar results. Furthermore, we wanted to explore if previous findings on correlations with emotional awareness measured by the manual English and French LEAS are replicable with the geLEAS. Therefore, we investigated the association of the geLEAS with sociodemographic data, self-report measures on emotional awareness and negative affect, the perception of affect in facial expression, and theory of mind functioning. We hypothesize that:

1. The geLEAS in comparison to hand scoring is a reliable method to evaluate emotional awareness yielding essentially the same score.
2. Female gender and higher educational attainment are positively associated with higher emotional awareness as measured by the geLEAS.
3. Emotional awareness as measured by the geLEAS is associated with a more accurate perception of affect in facial expressions and greater theory of mind capacity.
4. In contrast to self-reports, emotional awareness as measured by the geLEAS is not associated with negative affect.

## 2. Methods

### 2.1. Subjects

A German community sample consisting of 208 subjects (130 women and 78 men) was recruited by the Department of Psychosomatic Medicine and Psychotherapy at the Ruhr-University-Bochum, Germany in March 2022. The study sample had an average age of 32 years (*SD* = 12.54) and the vast majority of participants (175; 84%) completed at least 12 years of education (high-school diploma). Recruitment for participation took place via extensive public outreach and the use of social networking sites. We used flyers in paper and digital form to raise awareness of the study. Inclusion criteria were an age between 18 and 65 years and the absence of a mental disorder. Data were collected anonymously using SoSci Survey [(32), www.soscisurvey.de]. We relied on subjects' self-reports that the inclusion criteria were met. Participants received a compensation of 20*e*.

The study was approved by the institutional review board of the Medical Faculty, Ruhr-University, Bochum (No. 22-7485) and conducted according to the guidelines of the declaration of Helsinki. Participants were informed about the study's objective (assessment of perceived emotions and their impact on interpersonal relationships) and consent was obtained prior to participation. Due to the detail and length of the data collection, participants were able to pause questionnaires and continue the data entry at a later time.

### 2.2. Measures

#### 2.2.1. Sociodemographic questionnaire

Participants were given a series of questions about sociodemographic variables including questions about their level of academic achievement and occupational status.

#### 2.2.2. The Levels of Emotional Awareness Scale (LEAS 20) (12)

The LEAS consists of 20 scenarios that describe emotion-evoking interactions between two persons. Participants are asked to describe how they would feel (Self) and how the other person would feel (Other) in the scenario. Items are assessed using the aforementioned scoring rules. Each item is given a score between 0 and 5. A subject can achieve a score between 0 and 100 per protocol (sum score of all 20 items). Higher scores indicate higher emotional awareness.

#### 2.2.3. Toronto Alexithymia Scale (TAS-20) (33)

The TAS-20 is a self-report measure of alexithymia. The questionnaire contains 20 statements addressing the ability to recognize emotional states and distinguish them from bodily sensations. Self-ratings are made on a five-point Likert scale ranging from strongly disagree to strongly agree. We followed Carnovale's (34) recommendation and used the total score rather than the different subscales as a comparative value. In contrast to the LEAS, a higher score in the TAS-20 indicates more pronounced alexithymic traits.

#### 2.2.4. Perception of Affect Task (PAT), Subtask 2 (35)

Participants are shown 35 separate photographs of faces with specific expressions and are asked to indicate whether the label happy, sad, fear, anger, disgust, surprise, or neutral emotion best matches the expression in the face displayed. Each of the seven emotions is presented five times in a varying order.

#### 2.2.5. Mental State Stories (MSS) (36)

The MSS is a measure of cognitive theory of mind functioning. It presents 48 short stories and asks a true-false question about their content immediately thereafter. The measure is divided into four categories, each consisting of 12 short stories: two involving people, two involving objects; one in each category requires inferences and one does not. Inferences about people require inferences about mental processes (theory of mind). Higher scores reflect a greater ability to make pertinent mental or physical state attributions (31).

#### 2.2.6. Beck's Depression Inventory (BDI-II) (37) and Depression Anxiety Stress Scales (DASS-21) (38)

The BDI-II and DASS-II are two self-report measures of negative affect. Both measures consist of 21 questions that are each rated on a four-point scale. Higher scores in the BDI indicate greater depressive symptomatology. Higher scores on the DASS-21 indicate greater degrees of anxiety, depression, and tension/stress.

### 2.3. VETA

The Verbal Emotion in Text Assessment *Python* Package (VETA) is an open source, object-oriented *Python* package designed to both score and analyze LEAS survey data. Please refer to https://github.com/jacotay7/veta to obtain a copy of VETA.

VETA imposes a tiered class structure based on the inherent structure of a typical LEAS survey. Three main classes are defined within VETA: the *survey* class, the *respondent* class, and the *item* class. The item class is analogous to the nominal LEAS “item” and is generated using a single response to one of the questions on the LEAS questionnaire. The response must still be separated into sections which describe the “self” or the “other” if the user wishes to execute scoring methods that require that distinction (Please refer to section 2.4.1 for further information). The item class is responsible for prepping the responses for computer analysis, applying scoring methods when asked, storing the values of all scores that have been assigned to that item, and storing any auxiliary information that apply specifically to that item (e.g., number of unique words, vocabulary scores, etc.). The respondent class is comprised of a collection of items and corresponds to one complete LEAS questionnaire. Once the respondent object has been created, the user can choose to score all of the respondent's items, add auxiliary information about the respondent (e.g., age, gender, education, etc.), or export all of respondent's data to an array for further analysis. Lastly, the survey class is comprised of several respondents and consists of a survey or database including several complete LEAS questionnaires. Survey data can be read and written from/to spreadsheet files with a specified format; this functionality greatly reduces the barrier to entry for those with little programming experience. Additionally, the survey class contains methods that can apply basic data analysis techniques on the data such as computing correlations, plotting and linear fitting, and generating summary statistics. In addition to the aforementioned core functionality and class structure, VETA also provides the ability to load LEAS word lists (a list of words with their assigned LEAS score) from spreadsheets, as well as 14 different scoring methods (as described in this publication). A majority of the analysis presented in this paper was conducted using VETA and the analysis techniques used will be natively supported within the software.

### 2.4. Scoring

With the help of the pre-existing word list in English that was used for hand scoring and the English word list that was used for POES we created a new German word list consisting of 1,093 elements. Each element was assigned with a corresponding value. The LEAS protocols were scored in two different ways, manually and digitally. We decided to score more than one third of all protocols manually which were randomly selected with the constraint that gender and age were distributed equally in the manually scored sample. A fully-trained study team member scored 76 protocols (1,520 items). To ensure that the study team member who did the manual scoring was accurate and made subjective decisions according to the guidelines, he completed more than 10 h of training over a 4-week period with a scoring manual of the LEAS (15, 39) and received feedback on practice examples from an experienced LEAS rater. Inter-rater reliability was assessed by having an additional rater score 10 protocols (200 items). VETA searches for words in the items that match the word list. The program scored all 208 protocols, including the 76 protocols that were evaluated by the study team member. Protocols were not spell-checked before being scored by VETA. The participants' responses pertaining to Self and Other were assessed separately.

#### 2.4.1. Scoring methods

Consistent with the POES method of Barchard et al. (15), we implemented several methods in VETA to score the subjects' responses (Table 3):

- *Highest-4* calculates the sum of the four highest rated words of an item. This method does not differentiate whether Self or Other is feeling an emotion. The highest score to be achieved per item is 12, or 240 per protocol.
- *All-Sum* calculates the sum of all the values in an item. The highest achievable score for item and protocol is unlimited.
- *334* gives higher scores to items that contain several different emotion words than to responses in which identical words or phrases are used repeatedly. Method 334 searches for all words with a value of 3 in an item. If they are all identical, an item receives a final score of 3. If two words with a value of 3 are not identical, a score of 4 is assigned. If there are no words with a value of 3, the score for the item is the maximum value found in the list of scored words. The highest score to be achieved per item is 4, or 80 per protocol.
- *3345* is very similar to hand scoring. In this method, individual answers to the questions “How would you feel?” and “How would the other person feel?,” are examined to distinguish between emotions attributed to one self (Self) and the other person (Other). Self and other are scored separately according to the 334 method. An item is assigned a score of 5 if the scores for self and other are both 4; otherwise, the score for an item is the maximum of the self and other scores. The highest score to be achieved per item is 5, or 100 per protocol.
- *3345plus* is a scoring method that was newly implemented by VETA. The scoring method is identical to 3345 with the difference that an item is only assigned a score of 5, if the described emotions of Self and Other are not the same. 3345plus is the method closest to hand scoring. In the following analyses we emphasize this scoring method.

**Table 3:** Scoring methods.

**Scoring method**	**Explanation**	**Example score**
Highest-4	Calculates the sum of the four highest values	12
All-Sum	Calculates the sum of all of the values	15
334	Searches the wordlist for all values with a score of 1, 2, or 3. If two words with a score of 3 are not identical, an item score of 4 is assigned.	4
3345	Self and other responses are scored separately using the 334 method. An item score of 5 is given if self and other are assigned a score of 4; otherwise, the item score is the maximum of the self and other scores.	5
3345Plus^a^	Identical to the 3345 method with the additional constraint that to receive a 5, the wordlist entries found in the self and other sentences can not be identical	4
Word count	Counts every word of each item	31 (33^b^)
Vocabulary^a^	Counts every unique word of each item	25 (26^b^)
Time^a^	Measures the time that was needed to complete the LEAS	30 min

Example in German: “Mein ganzer Körper würde zittern. Ich würde mich nicht gut fühlen. Ich will so schnell wie möglich nachhause, da ich mich fürchte. Mein Partner fürchtet sich auch und will hier weg.”

English translation: “My whole body would shiver (1, physical sensation). I would not feel good (2, unspecific emotion). I want (3, specific emotion) to get home as soon as possible, because I am terrified (3, specific emotion). My partner is terrified himself and wants to go home.” To assess data entry pace, we excluded subjects who took breaks in between completing the LEAS (remaining *N* = 153).

^a^These scoring methods were newly implemented by VETA.

^b^The value in parenthesis represents the number of words of the English translation.

We were also interested in whether linguistic capabilities influenced emotional awareness as measured by the geLEAS. In addition to the aforementioned scoring methods, we also assessed the number of words used in a protocol (word count), the number of different words being used (vocabulary), and the time required to complete the LEAS (time, Table 3). For time, we excluded participants who took longer than one hour to complete the LEAS survey, assuming that they took a break during data entry.

### 2.5. Statistical analyses

For all correlation analyses with the geLEAS we used the scores from each subject's protocol. Please refer to Measures, LEAS 20 for more information on how a protocol is scored.

1. A Pearson correlation coefficient was computed to compare the scoring methods of the geLEAS and hand scoring. All scoring methods were tested for internal consistency using Cronbach's alpha. The mean difference in scores between the various scoring methods (334, 3345, 3345plus, and hand scoring) were calculated. We also compared the scores of the first and second rater using Pearson correlation.
2. We computed a Pearson correlation coefficient to assess the relation between emotional awareness as measured by the 3345plus and age, gender, education level, and whether subjects had children. A two-sided independent *t*-test was performed to determine if emotional awareness of men and women was significantly different.
3. We investigated the association between emotional awareness as measured by the 3345plus method and the results of the TAS-20, the PAT, and the MSS. We calculated the first order Pearson correlation coefficient, the correlation corrected for word count and vocabulary, and the correlation corrected for the time that was required to complete the LEAS.
4. We computed Pearson correlation coefficients to assess the relationship between 3345plus results and the self-report measures for negative affect (BDI-II and DASS-21). Furthermore, the relationship between the TAS-20 and negative affect was assessed using Pearson correlation.

## 3. Results

### 3.1. Internal consistency

All scoring methods showed excellent internal consistency. The all-sum scoring method had the highest internal consistency (α = 0.95; Table 4).

**Table 4:** Internal consistency of scoring methods using Cronbach's alpha.

**Scoring method**	**Cronbach's alpha**	**95% confidence interval**
Highest-4 (*N* = 208)	0.94	[0.93, 0.95]
All-Sum (*N* = 208)	0.95	[0.94, 0.96]
334 (*N* = 208)	0.89	[0.87, 0.91]
3345 (*N* = 208)	0.91	[0.89, 0.93]
3345Plus (*N* = 208)	0.91	[0.89, 0.93]
Hand scoring (*N* = 76)	0.90	[0.88, 0.92]

### 3.2. Correlation of scoring methods with hand scoring

**Figure 1:**
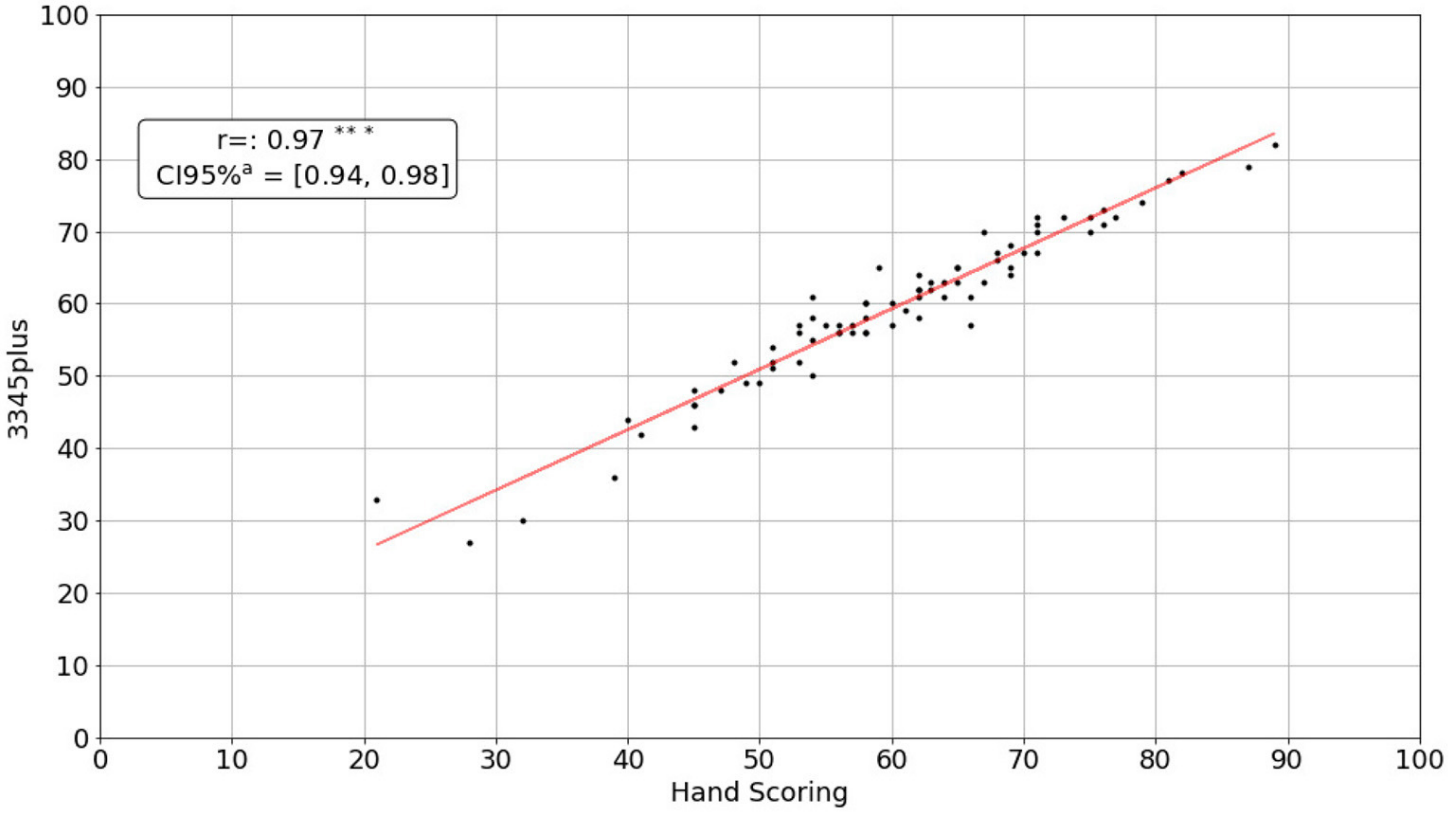
Correlation between 3345plus and hand scoring. ^*^P ≤ 0.05, ^**^P ≤ 0.01, ^***^P ≤ 0.001.

All scoring methods showed high correlations with hand scoring. The newly implemented 3345plus scoring method showed the numerically highest correlation (*r* = 0.97, *p* < 0.001, Figure 1). The scores of the 10 protocols that were analyzed by the first and the second rater were highly correlated (*r* = 0.97, *p* < 0.001). Additionally, we assessed the mean difference between the scores of each protocol by hand scoring and VETA's scoring methods. 3345plus replicated hand scoring most accurately. On average, the difference between hand scoring and 3345plus scoring was less than one point per protocol (Table 5).

**Table 5:** Correlations with hand scoring (*N* = 76).

	**Correlation**	**CI for correlation**	**Mean difference of**
			**respondents' score**
Highest-4	0.91^***^	[0.84, 0.95]	
AllSum	0.88^***^	[0.79, 0.93]	
334	0.94^***^	[0.9, 0.97]	1.29
3345	0.97^***^	[0.94, 0.98]	0.80
3345Plus	0.97^***^	[0.94, 0.98]	0.74
Second rater (*N* = 10)	0.97^***^	[0.94, 0.98]	0.10

The scores per protocol (the sum of the 20 item scores of every subject) were compared.

Second Rater and 3345Plus had a correlation coefficient of 0.94.

^*^P ≤ 0.05, ^**^P ≤ 0.01, ^***^P ≤ 0.001.

### 3.3. Correlation of emotional awareness with sociodemographic data

**Figure 2:**
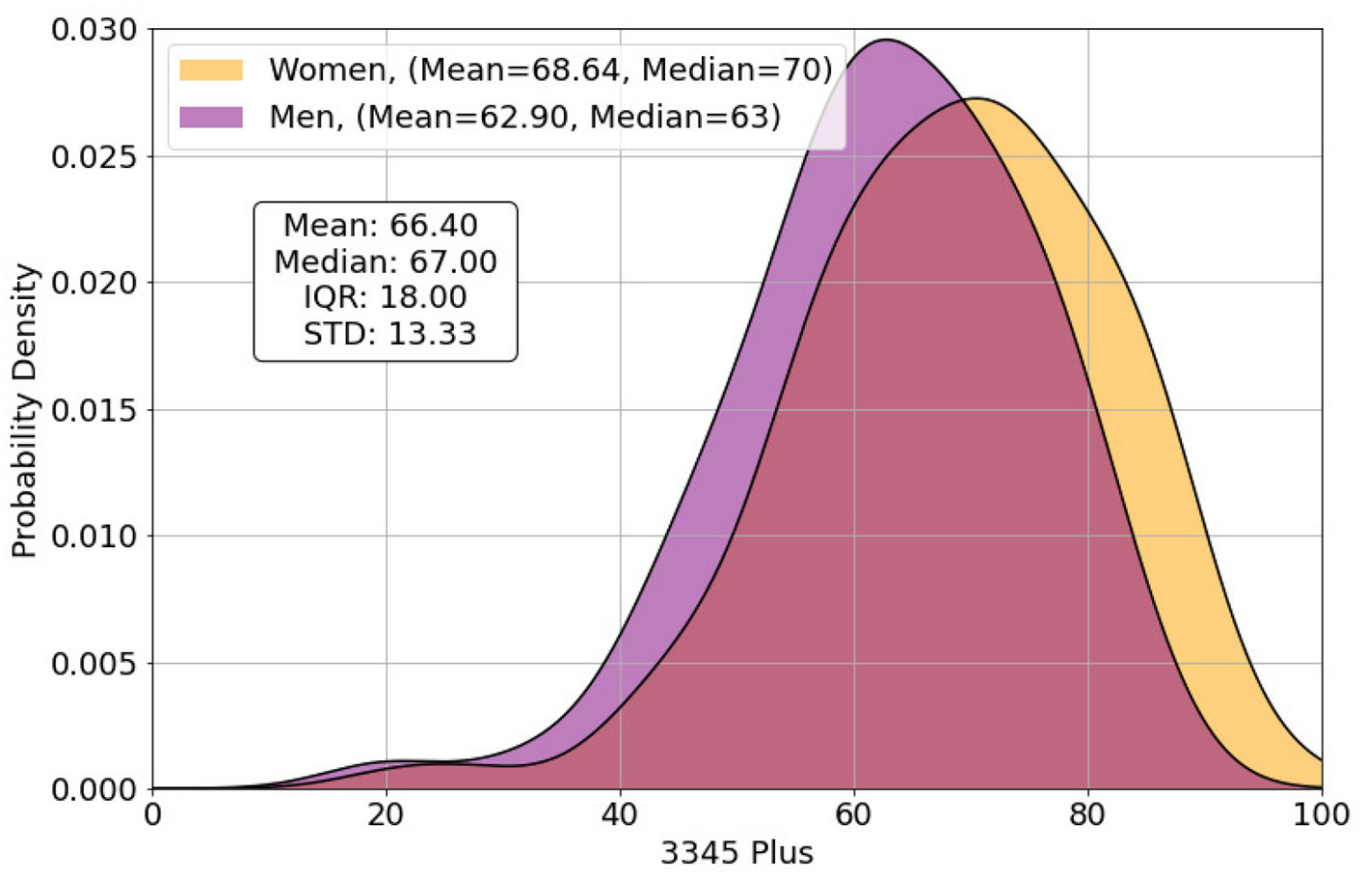
The distribution of the respondents' scores of the LEAS measured by the computer's 3345plus score.

As noted above, 208 subjects participated in this study; 130 women and 78 men. The median age of participants was 32 years (*SD* = 12.54). Please refer to the supplements for more sociodemographic information. As measured by the 3345plus method, participants reached an average score of 66.40 in emotional awareness. Women had a significantly higher emotional awareness than men [*t*_(168)_ = 3.10, *p* = 0.002; Figure 2]. Besides female gender (*r* = -0.23, *p* < 0.001), a higher academic degree (*r* = 0.34, *p* < 0.001), a younger age (*r* = 0.28, *p* < 0.001), and having children (44 subjects said they had children; *r* = 0.20, *p*=0.003) were each positively associated with higher emotional awareness (Table 6).

**Table 6:** 3345Plus correlation with sociodemographic data (*N* = 208).

	**Age^a^**	**Gender^b^**	**Education**	**Children^c^**
3345Plus	-0.23^***^	-0.23^***^	0.34^***^	0.20^**^
CI95%	[-0.35, -0.10]	[-0.35, -0.09]	[0.21, 0.45]	[0.07, 0.33]

^a^The subjects' average age was 32 years (SD = 12.54).

^b^Females: 130, males: 78.

^c^44 subjects said they had children.

^*^P ≤ 0.05, ^**^P ≤ 0.01, ^***^P ≤ 0.001.

### 3.4. Correlation of emotional awareness with psychometric test results

**Figure 3:**
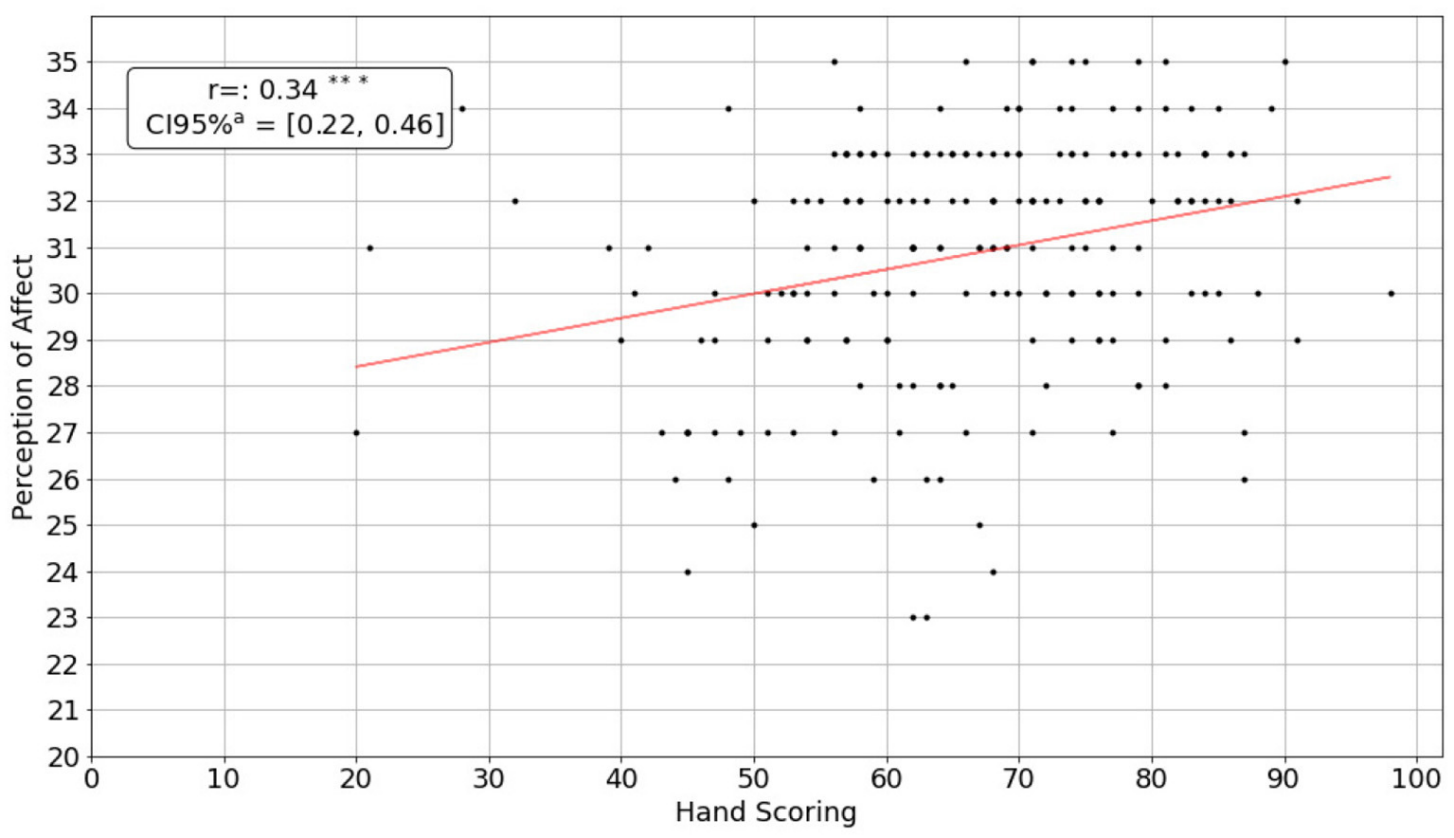
Correlation between 3345plus scores and the perception of emotions represented by facial expressions. ^*^P ≤ 0.05, ^**^P ≤ 0.01, ^***^P ≤ 0.001.

**Figure 4:**
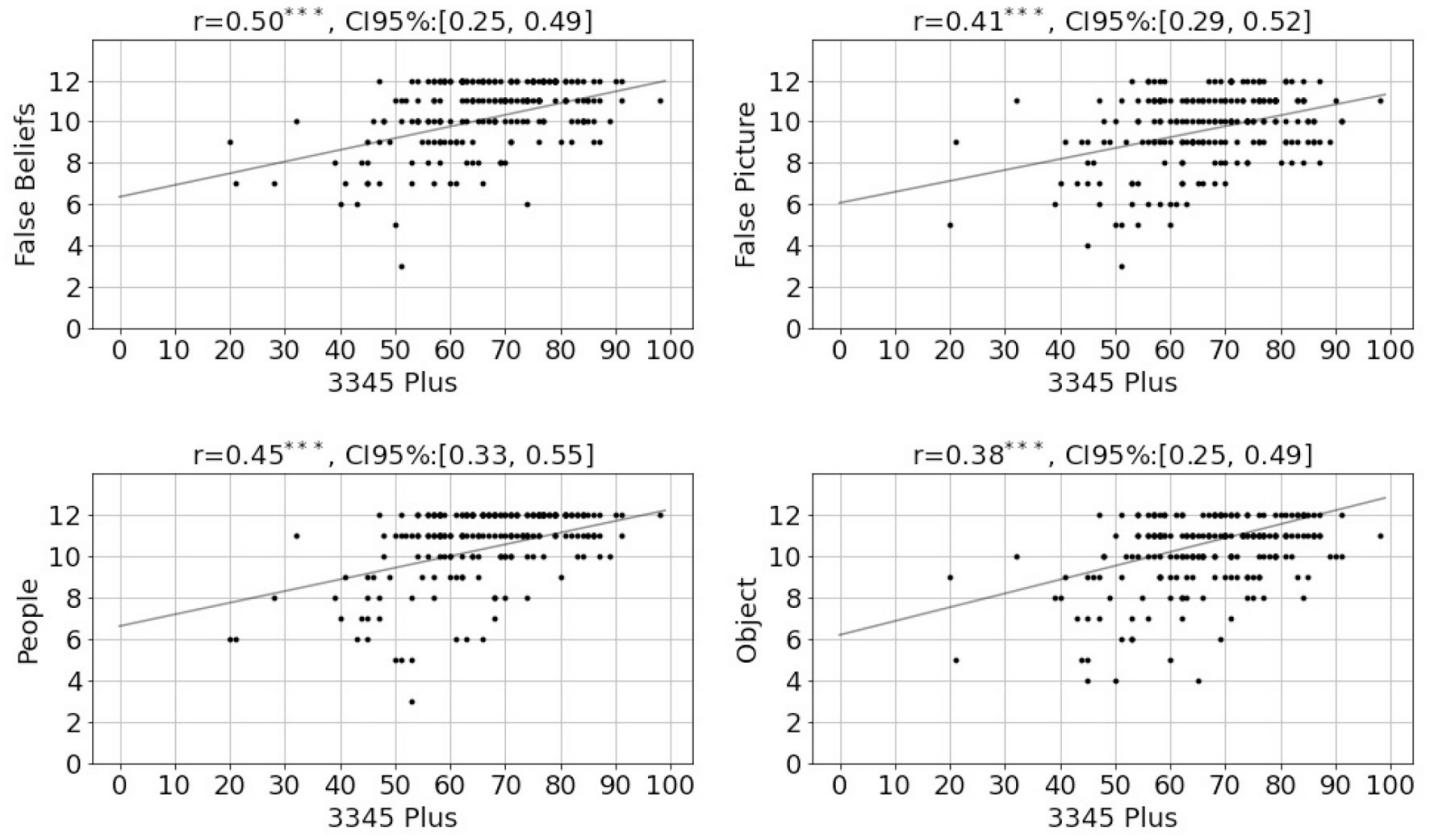
Relationship between the computer's 3345plus results and the different subcategories of the Mental State Stories. False beliefs, Inferences about people (Theory of Mind); False Picture, Inferences about objects; People, Facts about people; Object, Facts about objects. ^*^P ≤ 0.05, ^**^P ≤ 0.01, ^***^P ≤ 0.001.

Emotional awareness correlated with more accurate perception of emotions represented by facial expressions (*r* = 0.34, *p* < 0.001, Figure 3). Associations were maintained when correcting for word count and vocabulary (*r* = 0.28, *p* < 0.001) and also when correcting for the time required to complete the LEAS questions (*r* = 0.33, *p* < 0.001). Participants who had higher 3345plus scores, also scored higher in the Mental State Stories exercise (*r* = 0.51, *p* < 0.001). The association was the strongest when participants had to make inferences about mental processes (theory of mind, Figure 4).

The LEAS correlated positively with the TAS-20 (*r* = 0.23 *p* = 0.001). Emotional awareness and negative affect assessed by the BDI and the DASS showed no significant correlation (Table 7). There was a significant correlation between higher scores in the TAS-20 and negative affect measured by the BDI (*r* = 0.31, *p* < 0.001) and the DASS (*r* = 0.50, *p*= < 0.001, Table 8).

**Table 7:** Correlations of scoring methods with psychometric test results.

	**PAT**	**MSS**	**TAS-20**	**BDI**	**DASS**	**Word count**	**Vocab**	**Time**
	**(*N* = 208)**	**(*N* = 202)**	**(*N* = 208)**	**(*N* = 205)**	**(*N* = 207)**			
3345Plus	0.34^***^	0.51^***^	0.23^**^	0.00	0.10	0.60^***^	0.59^***^	0.40^***^
	0.28^a***^	0.45^a***^	0.20^a**^	-0.06^a^	0.10^a^		
	0.33^b***^	0.45^b***^	0.24^b**^	-0.08^b^	0.10^b^		
CI95%	[0.22, 0.46]	[0.40, 0.61]	[0.09, 0.35]	[-0.14, 0.14]	[-0.03, 0.24]	[0.50, 0.68]	[0.5, 0.68]	[0.26, 0.53]
334	0.37^***^	0.55^***^	0.27^***^	-0.03	0.11	0.50^***^	0.51^***^	0.36^***^
	0.31^a***^	0.49^a***^	0.25^a***^	-0.09^a^	0.11^a^		
	0.36^b***^	0.51^b***^	0.29^b***^	-0.10^b^	0.11^b^		
CI95%	[0.24, 0.48]	[0.45, 0.64]	[0.14, 0.39]	[-0.17, 0.10]	[-0.03, 0.24]	[0.39, 0.60]	[0.40, 0.60]	[0.22, 0.49]
AllSum	0.27^**^	0.40^***^	0.18^**^	0.07	0.10	0.76^***^	0.72^***^	0.47^***^
	0.20^a**^	0.32^a***^	0.16^a*^	0.01^a^	0.08^a^		
	0.25^b**^	0.35^b***^	0.19^b*^	-0.04^b^	0.10^b^		
CI95%	[0.14, 0.39]	[0.27, 0.51]	[0.04, 0.31]	[-0.06, 0.21]	[-0.04, 0.23]	[0.70, 0.82]	[0.65, 0.78]	[0.33, 0.58]
Highest4	0.33^***^	0.49^***^	0.24^***^	0.03	0.11	0.66^***^	0.66^***^	0.45^***^
	0.26^a***^	0.42^a***^	0.23^a***^	-0.04^a^	0.10^a^		
	0.30^b***^	0.42^b***^	0.26^b**^	-0.06^b^	0.11^b^		
CI95%	[0.20, 0.45]	[0.38, 0.59]	[0.11, 0.37]	[-0.11, 0.16]	[-0.05, 0.26]	[0.58, 0.73]	[0.57, 0.73]	[0.32, 0.57]

^a^Variables are tested for partial correlation using Length and Vocab as control variables.

^b^Variables are tested for partial correlation using Time as the control variable.

For this corrective measure we excluded subjects who took longer than an hour to complete the LEAS assuming that they took breaks in between (remaining *N* = 153).

^*^P ≤ 0.05, ^**^P ≤ 0.01, ^***^P ≤ 0.001.

**Table 8:** Correlation of the TAS 20 with psychometric test results.

	**PAT**	**MSS**	**BDI**	**DASS**
	**(*N* = 208)**	**(*N* = 202)**	**(*N* = 206)**	**(*N* = 207)**
TAS-20	0.15^*^	0.31^***^	0.31^***^	0.50^***^
CI95%	[0.02, 0.28]	[0.17, 0.43]	[0.18, 0.43]	[0.39, 0.59]

^*^P ≤ 0.05, ^**^P ≤ 0.01, ^***^P ≤ 0.001.

## 4. Discussion

In this study, we present the geLEAS, the first non-English electronic evaluation program of the LEAS. The computerized evaluation generated results that were very similar to those of a well-trained human LEAS rater, demonstrating the reliability and validity of the geLEAS. All computerized scoring methods showed a strong correlation with hand scoring. Reliability was demonstrated by the high internal consistency of all scoring methods and the high interrater correlations. Given its high correlation with hand scoring, we focused primarily on the 3345plus method in our investigations. The other scoring methods also show promising results. Furthermore, these methods can be used to evaluate the emotional content of text unrelated to the LEAS. The all-sum method for example could be applied on any open-ended text and provide a score that represents emotional functioning. Thus, the advent of the geLEAS considerably expands the capacity to do research on emotional awareness including in psychiatric contexts.

We wanted to explore if previous findings on the LEAS could be replicated in a German-speaking sample using computerized evaluation. When investigating the relation between emotional awareness and sociodemographic data, we observed that women had significantly higher emotional awareness scores than men. This phenomenon has been demonstrated in previous research. Barrett et al. (30) reviewed findings on sex differences in the complexity and differentiation of emotional experience and found that in seven different subsamples, ranging in age, education, and culture, women always showed higher emotional awareness than men. There is still an open discussion about where this stable and generalizable gender difference in emotional awareness comes from. One frequently cited hypothesis is that, due to different socialization processes between boys and girls, women have better access to their emotional knowledge or are more willing and interested in using that access (40–42). Another hypothesis, that is not mutually exclusive, is that there are genetically based sex differences in emotional attunement related to the greater role of females in caring for the young in our evolutionary history (43). Similar cultural learning explanatory approaches are used to explain the age effect on emotional awareness. In prior generations, learning how to access the emotional repertoire and being able to accurately identify emotions might not have been a major focus in socialization and personal development (28), i.e., the observed age differences are cohort effects due to differing social influences in different time periods.

The positive association of the geLEAS with education, which has previously been demonstrated on the LEAS (14, 28, 29), has been explained by the greater verbal ability associated with higher levels of educational attainment. Verbal ability is closely connected to the ability to describe emotions in a varied and diversified way. This explanation raises the issue whether associations of emotional awareness with other psychometric measures should be corrected for word count and vocabulary. Given that higher scores on the LEAS are assigned when more emotion words are used, giving more lengthy answers might inherently lead to higher emotional awareness scores (2). Recent studies suggest, however, that verbal abilities do not simply constitute a “read out” (comparable to printing a finished document) of emotional experiences, but in fact also play an important role in shaping them (44). Thus, verbal ability and verbal expression of emotional terms would not only be a positive predictor, but a central element of emotional awareness. Given the inseparability of awareness and reporting (45), Lane and Smith (2) suggest that controlling for word count and vocabulary may remove substantive variance from the phenomenon under investigation. An alternative approach is our newly implemented control variable “time for completing the LEAS,” which provides a method for controlling for the subjects' motivation without removing linguistic ability from the measure of emotional awareness. It is notable that our results did not change substantively when controlling for this variable. Tracking time to completion is an added benefit of digital administration of the LEAS.

Regardless of whether one examines the first order correlation or the association controlled for word count, vocabulary, or time to completion, our findings indicate a significant association between emotional awareness measured by the geLEAS and the ability to accurately perceive facial expressions. Lane et al. (14) made a similar observation previously. This association strengthens the concurrent validity of the geLEAS. To accurately identify emotional expressions in human faces requires a mental representation of emotion concepts that is closely related to the ability to anticipate emotions in hypothetical situations. Similarly, emotional awareness measured by the geLEAS was associated with a better ability to infer mental states in other people. This finding was previously demonstrated by Lane et al. (31). The LEAS targets a person's ability to create mental representations of hypothetical emotional states of oneself and others. This ability appears to be a domain-specific expression of the domain-general theory of mind function (46).

Our results support the widespread observation that the LEAS and the TAS-20 measure partially overlapping concepts of emotional functioning (47). geLEAS and TAS-20 scores were positively correlated. As higher scores in the TAS-20 indicate a greater number of alexithymic characteristics, one might expect the opposite. Although most studies report a negative correlation between the two scales (47), several researchers reported a positive correlation similar to ours (7, 48–50). It has been suggested previously that the content of the TAS-20 items may lead subjects to give alexithymic responses even though they are not alexithymic (29). For example, in a healthy sample, subjects who are better able to access their own emotions might also be more inclined to report on a perceived inability to describe how they feel in certain emotion evoking situations. This is supported by the counter-intuitive observation that TAS-20 was positively (not negatively) correlated with MSS. This conclusion that self-report may be an inaccurate indicator of the variable being assessed could further explain the association of TAS-20 and negative affect. Individuals who experience a flattening of affect could endorse items due to low motivation or energy (“I am able to describe my feelings easily”) or a negative self-concept (“It is difficult for me to find the right words for my feelings.”). In line with our hypothesis, emotional awareness measured by the geLEAS was not associated with negative affect. For a further discussion of similarities and differences between the two measures see Lane et al. (51).

There are limitations to this study. Our data were collected on a healthy community sample. The average age of our sample was a relatively young 32 years of age and subjects on average had a high level of educational attainment. Although these factors do not compromise the functioning of the geLEAS as a valid emotion assessment tool, the feasibility of using the geLEAS on clinical samples remains to be verified. A key assumption in the use of the geLEAS in psychiatric research is that patients will be able to complete it successfully. It is assumed that the participants under investigation are familiar with data entry on digital media. Digital data collection can be a cognitive challenge, especially for clinical samples (52). Clinicians and researchers should keep this in mind when considering the use of the geLEAS in patients. Research shows, however, that with assistance even patients with serious mental illnesses such as schizophrenia can successfully complete digital inventories. At this point, it should also be addressed that although the geLEAS saves time and labor during the evaluation, the survey is still rather time-consuming for the subjects (1–2 min per item). A validation of the automatic evaluation of the 10-item LEAS version, whose validity and reliability in manual form has already been reported (2, 27), should be performed in a future study. Another limitation is that hand and computer scoring was completed in only 76 protocols. In comparison to sample sizes used in the creation of the first version of the eLEAS in English computerized approach, this number is quite sufficient (15). However, additional studies comparing manual scoring and computer scoring of the geLEAS should be explored. Lastly, although we have tried to make the installation and operation of the software program as simple and user-friendly as possible, basic programming skills are still required for implementation. VETA is a work in progress and we will work on further developing the user experience.

With the help of the geLEAS, emotional awareness of patients and study participants can now be assessed automatically in English and German (https://eleastest.net). We encourage other scientists to create additional computerized versions of the LEAS in more languages. VETA has been designed so that with an appropriate word list this transfer can be made quickly and conveniently. Our results indicate that the manual and computerized German LEAS measure the same concept of emotional awareness as assessed in English. The geLEAS can therefore potentially facilitate and accelerate research on the development, onset, and clinical course of mental disorders. Lastly, we encourage clinicians to integrate the geLEAS in the clinical setting. Particularly the 3345plus method can quickly and validly provide conclusions about the emotional functioning of participants. Thus, it has the potential to become an important variable in predicting disease progression in patients with mental health disorders.

## Data availability statement

The raw data supporting the conclusions of this article will be made available by the authors, without undue reservation.

## Ethics statement

The studies involving human participants were reviewed and approved by the institutional review board of the Medical Faculty, Ruhr-University, Bochum (No. 22-7485). All participants received written information about the study online and had to give informed consent before participating.

## Author contributions

JH, JT, RL, and JD-H did the main conceptualization. JH and JT conducted the statistical analysis. JH and RL did the interpretation of the data. JH, JT, and RL wrote the first draft of the manuscript. CS-W was the second rater for the manual scoring of the LEAS. MR helped collecting the LEAS data. SH, NO, and JA supported the conceptualization, designed, and supervised and supported the data collection. All authors did critical review, commentary, and revision. All authors contributed to the article and approved the submitted version.
