# Supplementary material for: Development and validation of a computer program for measuring emotional awareness in German—The geLEAS (German electronic Levels of Emotional Awareness Scale)
Source: Front Psychiatry. 2023 Mar 23;14:1129755. doi: 10.3389/fpsyt.2023.1129755 (PMC10076697; doi:10.3389/fpsyt.2023.1129755)
Supplement: Supplementary file 1 [file Data_Sheet_1.PDF]

1 SUPPLEMENTARY MATERIAL

Table S1. Distribution of the highest level of educational attainment of the study sample

Educational attainment	Number of subjects (N=208)	Percentage
No school diploma	2	1%
Primary education	1	0.5%
Secondary education first stage	22	10.6%
Secondary education second stage	81	38.9%
Post-secondary non tertiary education	12	5.8%
First stage of tertiary education	90	43.3%

Note: In the definition of educational attainment, we were guided by the International Standard Classification of Education (ISCED-97; Schneider (2008)

Table S2. Distribution of the occupational status of the study sample

Occupational status	Number of subjects (N=208)	Percentage
Not employed	6	2.9%
Retired	6	2.9%
In training	103	49.5%
Employed (full or part time)	93	44.7%

REFERENCES

Schneider, S. L. (2008). *The international standard classification of education (ISCED-97): An evaluation of content and criterion validity for 15 European countries*
